# Formation Mechanism of Polypyrrole-Coated Hollow Glass Microspheres (PPy@HGMs) Composite Powder

**DOI:** 10.3390/ma17225595

**Published:** 2024-11-15

**Authors:** Yao Du, Jianfeng Zhang, Ning Wang, Lei Liu, Jun Wang, Yahui Liu, Gaiye Li, Chuanhua Xu

**Affiliations:** 1 College of Materials Science and Engineering, Hohai University, Nanjing 210098, China; dyjy19990920@163.com (Y.D.); 17364303320@163.com (N.W.); ligaiye@126.com (G.L.); 2 Sinosteel Maanshan New Material Technology Co., Ltd., Maanshan 243000, China; llei_89@163.com (L.L.); wangjun4611@sina.com (J.W.); kyliuyahui@126.com (Y.L.); chuanhuaxu@163.com (C.X.)

**Keywords:** hollow glass microspheres (HGMs), polypyrrole (PPy), lightweight, capping, silane coupling agent

## Abstract

Coating conductive nanoparticles onto the surface of hollow glass microspheres (HGMs) is essential for broadening their applications. However, the low density and high specific surface area of HGM powders, along with the thin walls of the cavity shells and poor surface adhesion, pose challenges for the uniform attachment of functional particles. In this study, we developed a novel integrated process that combines flotation, hydroxylation, and amination pretreatment for HGMs with in situ surface polymerization to achieve a uniform coating of polypyrrole (PPy) on the surface of HGMs. We explored the corresponding growth process and coating mechanism. Our findings indicate that the amount of coating, particle size, and uniformity of PPy on the surface of HGMs are significantly influenced by the pretreatment and the in situ polymerization time, as well as the microspheres/pyrrole feedstock ratio. The in situ polymerization on the surface of HGMs resulted in a uniform encapsulation of spherical PPy, with the average particle size of PPy-coated HGMs (PPy@HGMs) increasing by 14.60% compared to the original HGMs. The elemental nitrogen in the PPy@HGMs primarily exists in the form of C-N and N-H bonds. This study demonstrates that the surface functional groups of HGMs engage in chemical bonding and interactions with PPy molecules. Mechanistic analysis reveals that the hydroxyl and amino groups enriched on the surface of the pretreated HGMs serve as activation centers, facilitating the uniform enrichment of pyrrole monomers and promoting chain growth polymerization of the conjugated chain through nucleophilic and electrophilic interactions with the subamino groups in the pyrrole ring. Additionally, the reaction between the Lewis acid properties of PPy and the Lewis-type electron-donating amino groups in KH550 fosters strong bonding and the formation of a robust interface.

## 1. Introduction

HGMs are made mostly of borosilicate and have a hollow, thin-walled spherical structure. They have great qualities such as low density, high strength, thermal insulation, mobility, and stability [1,2,3]. HGMs were initially utilized in aerospace, oil and gas development, buoyancy materials, and other advanced technologies. With the advancement of science and technology in China and the realization of industrial-scale mass production, HGMs have begun to see widespread application in civil sectors, including paints, cements, emulsified explosives, and adhesives [4,5,6,7].

With the increasing depth of research into the development and application of HGMs, surface modification technology has become increasingly important [8,9,10]. On the one hand, when high-performance HGMs are incorporated into a polymer matrix as fillers, compatibility issues often arise due to the significant difference in polarity between the two materials. Therefore, modification treatments are frequently necessary to enhance compatibility prior to incorporation [11,12,13]. On the other hand, HGMs possess a unique hollow, thin-walled structure that not only effectively reduces the density of composite materials but is also relatively inexpensive. Additionally, by coating a layer of specialized functional materials on their surface, HGMs can form a core–shell hollow structure, resulting in a new material with unique properties such as wave absorption, flame retardance, adsorption, and reflectivity [14,15,16]. For example, in the field of photocatalysis, pure TiO_2_ has garnered significant attention from researchers due to its excellent stability, non-toxicity, and low preparation cost. However, its practical applications are limited by factors such as light efficiency and overall cost. The photocatalytic effectiveness of TiO_2_ can be significantly enhanced by coating it onto the surface of microspheres, forming a spherical shell-like structure. This approach leverages the incident and reflective properties of the microspheres to optimize light interaction [17,18]. In the field of electromagnetic shielding, HGMs, despite their average electromagnetic shielding performance, possess several advantageous characteristics, including low density, small particle size, hollowness, lightweight nature, high strength, heat insulation, and chemical stability. These properties make them an excellent substrate for the preparation of electromagnetic shielding materials [19].

In the conductive modification of HGMs, intrinsically conductive polymers such as polycarbazole, polyaniline, and polypyrrole [20,21,22,23] are selected as fillers; these polymers are able to carry conductivity on their own due to their conjugated structure, and they usually have low density and good corrosion resistance, which makes them well suited for use as fillers in electromagnetic shielding composites. In addition, these polymers can be blended with matrix materials, such as silicone rubber [24,25,26], to form composites that can be easily processed and molded while maintaining good electromagnetic shielding effectiveness. Traditionally, conductive magnetic metal fillers, such as iron, cobalt, and nickel, along with their oxides, have garnered the attention of researchers [27,28,29]. Although their performance is good, the main disadvantage is an increase in the density of materials after compounding, which significantly limits their range of applications.

However, modified HGMs can address this issue. Bu et al. [28] employed the HCl+NH_4_F method to roughen the surfaces of HGMs, subsequently plating cobalt nanoparticles onto the microspheres using a chemical plating process. Imran et al. [30] coated carbon nanotubes onto HGMs using chemical vapor deposition (CVD) to investigate the effect of processing temperature on the formation of carbon nanotube materials on these microspheres. Surface modification of HGMs is essential for expanding their application scope, with research in this field growing more diverse. The main methods explored include acid and alkali etching, the use of coupling agents, polymer grafting, and surface coating. For in situ polymerization on non-metallic substrates like HGMs, pretreatments are necessary to create active groups that initiate polymerization on the microsphere surface, leading to a uniform and dense coating. There is a pressing need to optimize these processes and clarify the mechanisms behind HGM modification.

In this work, PPy-coated HGMs were utilized as a case study to develop the coating process for conducting polymer nanoparticles on the surface of HGMs and to investigate the associated mechanisms. Techniques such as flotation (as shown in Figure S1), hydroxylation, amination, and optimization of coating process parameters were employed to obtain PPy@HGMs with varying amounts of HGMs coating. Scanning electron microscopy and laser particle size analysis were used to assess the apparent properties, micro-morphology, quality changes, coating rates, and particle size distribution of HGMs before and after modification. Additionally, these methods were employed to explore the mechanism of the in situ polymerization process for coating HGMs.

## 2. Experimental Materials and Methods

### 2.1. Experimental Materials

The HGMs used in this experiment were produced using the precursor method. The primary chemical composition was borosilicate, and the appearance was that of a white powder with good fluidity. The density of the microspheres was 0.38 g/cm^3^, the compressive strength was 20.0 MPa, the average particle size was 43.1 μm, the water content was 2.0%, and the pH was alkaline, approximately 8.50.

### 2.2. Treatment Process

**Figure 1:**
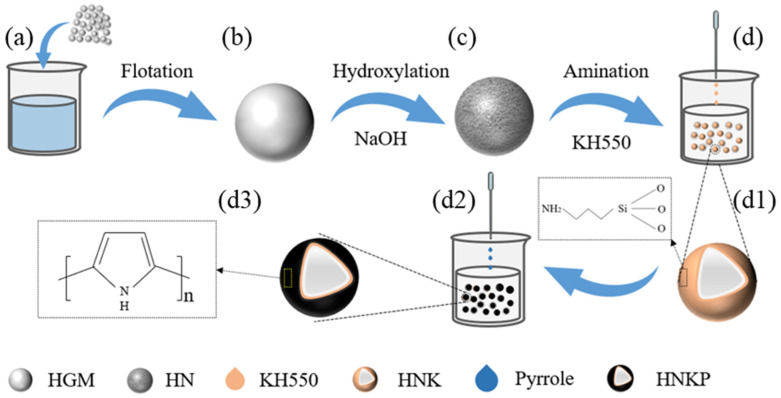
Diagram of the experimental process of PPy@HGMs composite powder preparation. (**a**,**b**) flotation (**b**,**c**) hydroxylation (**c**,**d**) amination (**d1**) chemical composition of d (**d2**,**d3**) coated polypyrrole.

The flowchart for the preparation of PPy-coated HGM powders is illustrated in Figure 1. Initially, the microspheres undergo pretreatment to achieve surface-aminated microspheres, which involves multiple flotation, hydroxylation, and amination processes. This is followed by the introduction of pyrrole monomers through in situ polymerization, resulting in the formation of the composite powder [31]. Specific descriptions are given below (the solutions used are shown in Table 1).

(1) Flotation treatment.

A number of HGMs were placed in a deionized water solution at room temperature with magnetic stirring for 10 min. The ratio of HGMs to deionized water was 1:10, followed by ultrasonic treatment for 10 min, washing and filtration, and drying in the oven for 8.0 h.

(2) Hydroxylation treatment.

To begin, a 0.5–1.5 mol/L NaOH solution was prepared. An amount of 10.0 g of flotation HGMs was added to 200 mL of the solution and heated to 50 °C. The reaction was carried out by reflux stirring in a water bath at 400 rpm for 2.0 h and then allowed to stand at room temperature for 30 min afterward. After flotation, it was washed to neutrality with deionized water, filtered, and dried in an oven for 8.0 h to yield hydroxylated HGMs.

(3) Amination treatment.

Before the HGMs are coated with polypyrrole, the surface must be activated by grafting amino groups to direct the in situ polymerization of pyrrole monomer on the surface. First, ethanol and deionized water were mixed at a mass ratio of 1:3, and the ethanol solution was obtained by stirring. To prepare the activation solution, 1.0 mL of silane coupling agent KH550 was added to the ethanol solution with a pipette and thoroughly mixed. Subsequently, 10.0 g of hydroxylated HGMs was weighed and immersed in 200 mL of activation solution at 80 °C. The reaction was carried out by reflux mechanical stirring in a water bath at a speed of 400 rpm for 2.0 h, after which the reaction was allowed to stand at room temperature for 30 min before being washed with deionized water to neutrality after flotation, and then pumped, filtered, and dried in an oven for 8.0 h to obtain the amino-carbonated HGMs.

(4) Coated polypyrrole

To begin, a solution of 5-sulfosalicylic acid at a concentration of 1 mol/L, FeCl_3_ at a concentration of 1 mol/L, and an ethanol solution with a mass ratio of ethanol to deionized water of 10:1 were prepared. A specific amount of pyrrole monomer was weighed and 50 mL of 5-sulfosalicylic acid was prepared to create the polymerization stock solution. Subsequently, 2.0 g of aminated HGMs was placed in 400 mL of polymerization stock solution, mechanically stirred for 10 min at 400 rpm in an ice-water bath; then, 50 mL of Fecl_3_ with a concentration of 1 mol/L was added, and the reaction was mechanically stirred under reflux for 1.0–3.0 h at 400 rpm in an ice-water bath. After the stirring was completed, the reaction was allowed to remain for 30 min at ambient temperature before being neutralized with deionized water. After flotation, it was neutralized with deionized water, filtered, and oven-dried for 8.0 h to produce polypyrrole-coated HGMs.

### 2.3. Characterization

An electrically heated constant temperature drying oven (AUY-120, SHIMADZU, Tokyo, Japan) was used to dry the HGMs. A density meter (JW-M100, Jingwei Gaobo Technology, Beijing, China) was used to test the density of the materials. A scanning electron microscope ((S-4800, Hitachi, Honshu Island, Japan) was utilized to examine the microscopic morphology of hydrogel microspheres (HGMs) and their composites. The surface chemical composition and bonding of HGMs, both before and after modification, were analyzed using X-ray photoelectron spectroscopy (MiniFlex 600, Rigaku, Tokyo, Japan). A metallographic microscope (6XB-PC, Optical Instrument Factory, Shanghai, China) was used to observe the microscopic morphology of the composites. A laser diffraction particle size analyzer (Mastersizer 3000, Malvern, Worcestershire, UK) was employed to assess the changes in the particle size distribution of HGMs before and after modification. The density of the composites was measured using a density balance (AUY-120, SHIMADZU, Tokyo, Japan). A thermogravimetric analyzer (TG 209 F Tarsus, Naichi, Bavaria, Germany) was used to obtain heat loss graphs.

In this work, the surface hydroxyl content of HGMs was characterized using a titration method [32]. The procedure was as follows: a specific amount of HGM sample was weighed, and then 80 mL of aqueous sodium hydroxide solution with a concentration of 0.05 mol/L was added to a conical flask. The flask was sealed and stirred for 24 h. The inorganic compounds were separated by centrifugation, and 10 mL of the clear supernatant was collected and titrated with A mL of aqueous hydrochloric acid solution at a concentration of 0.05 mol/L until neutralization was achieved. The above steps were repeated using a blank solution, resulting in B mL of hydrochloric acid required for neutralization. The surface hydroxyl content of the HGMs sample was calculated as follows:

(1) $$X=\frac{(B-A)\;\text{×}\;0.05\;\text{×}\;8}{W}$$

X is the number of surface hydroxyl groups per unit weight of HGMs in mM/g.

## 3. Results and Discussion

### 3.1. HGMs Surface Pretreatment

#### 3.1.1. Flotation

Through the characterization of the microscopic morphology of the HGMs and the testing of the flotation rate, it can be found that the particles of the original microspheres are not completely hollow structures of the regular sphere. There are still a large number of defects, such as broken, un-foamed particles, holes, irregular shapes and other defects in the microspheres [33]. The existence of these defects significantly affects the subsequent modification of HGMs and the performance of electromagnetic shielding materials, so it is necessary to carry out the flotation treatment of HGM powder. The mass of HGMs before and after flotation is reduced by 2.37 g, the flotation rate is increased from 76.42% to 96.26%, the average particle size is increased from 51.536 μm to 54.538 μm, and the density is reduced from 0.3841 g/cm^3^ to 0.3111 g/cm^3^. These data show that the flotation treatment can effectively remove the defective particles in the raw powder of microspheres and reduce the density of HGMs [34].

SEM was used to characterize the morphology of HGMs before and after flotation, as shown in Figure S2. From Figure S2(a1,b1), it can be found that after the HGMs underwent flotation, the smaller particle size and microspheres with defects basically disappeared, which is because the un-foamed small-particle-size microspheres and microspheres with defects have a high density and sink underwater. The flotation process removes all the submerged microspheres, leaving behind regular and full-shaped HGM spherical particles. Figure S2(a2,b2) mainly characterizes the microscopic morphology of defective and normal microspheres. Common defective HGMs are mainly characterized by incomplete foaming, holes, crushing, irregular shape, etc. The causes of defective microspheres may include raw materials, production processes, temperature, equipment, and other issues.

#### 3.1.2. Hydroxylation

In this experiment, NaOH solution was used to hydroxylate HGMs [35,36]. On the one hand, the alkaline solution can roughen the surface of HGMs, which is convenient for the subsequent activation treatment; on the other hand, NaOH solution can undergo alkali-catalyzed hydrolysis reaction with the silica–oxygen bonding in the HGMs, which generates sodium silicate (Na_2_SiO_3_). The sodium silicate contains hydroxyl groups (−OH), so that more hydroxyl groups appear, thus increasing the hydroxyl content on the surface of the microspheres, which is beneficial for the subsequent HGMs to pick up amino groups. The reaction formula is as follows:

2NaOH + SiO_2_→Na_2_SiO_3_ + H_2_O (2)

**Figure 2:**
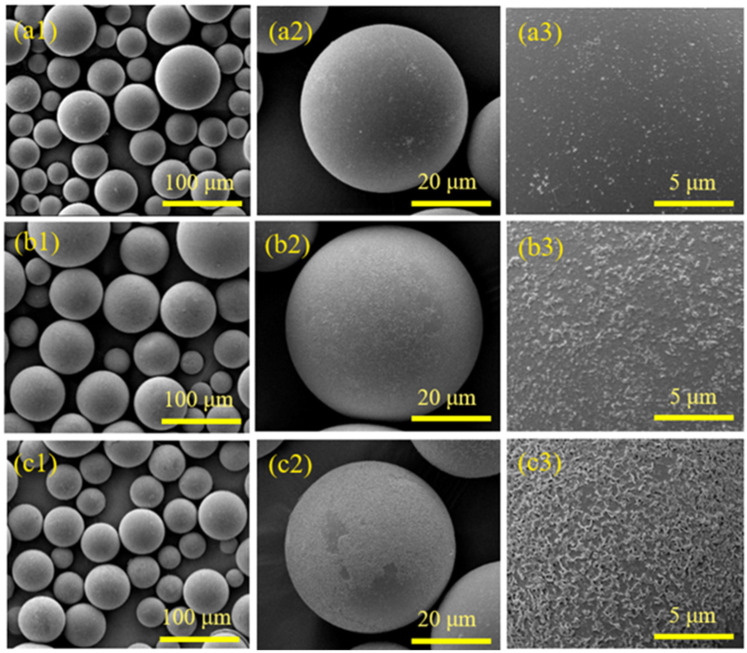
SEM images of HGM pretreatment with different concentrations of NaOH solution: (**a1**–**a3**) raw powder; (**b1**–**b3**) 0.75 mol/L; (**c1**–**c3**) 1.50 mol/L.

Figure 2 shows the microscopic morphology of HGMs after treatment with different concentrations of NaOH solution. When not treated with NaOH solution, the surface of HGMs did not show too many bumps and was very smooth with only a few white particles. When the concentration of NaOH solution was 0.75 mol/L, a lot of white flocculent aggregates on the surface of HGMs could be clearly seen through the SEM, and the surface of HGMs in Figure 2(b1,b2,b3) appears rougher compared with that in Figure 2(a1,a2,a3), which is caused by the alkali-catalyzed hydrolysis reaction of the NaOH solution with the silica–oxygen bonding on the surface of HGMs, generating Na_2_SiO_3_. When the concentration of NaOH solution is 1.50 mol/L, there are obvious etching traces on the surface of hollow microspheres and even a few tiny holes, and the reaction generated more Na_2_SiO_3_ attached to the surface of HGMs.

**Figure 3:**
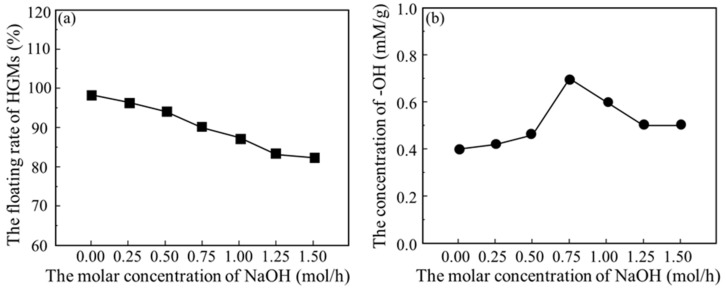
Effect of NaOH solution concentration on (**a**) floating rate and (**b**) hydroxyl content of HGMs.

Figure 3 shows the effect of NaOH solution on the flotation rate and hydroxyl content of HGMs. When the concentration of NaOH solution was gradually increased, the flotation rate of HGM powder gradually decreased from about 97.0% to about 83.0% of the original powder of microspheres. This is because after the reaction between NaOH solution and HGMs, the surface of the microspheres became rougher due to the chemical reaction, resulting in many stress weak points, and the compressive strength decreased. Coupled with the need for constant mechanical stirring of the solution during the hydroxylation process, this led to the breakage of some of the microsphere particles. When the concentration of NaOH solution was gradually increased, the hydroxyl content on the surface of HGM powder showed a tendency to rise and then decrease and reached the maximum value of 0.68 mM/g at the concentration of NaOH solution of 0.75 mol/L. The reason is thought to be that the increase in NaOH solution concentration in the initial stage accelerated the hydrolysis reaction of the silica–oxygen bond, which led to the emergence of more hydroxyl (−OH) on the surface. However, when the surface became covered by a large number of hydroxyl groups, further elevation of the NaOH solution concentration was limited by the saturation of the surface, leading to the stabilization or decrease in the hydroxyl content.

#### 3.1.3. Amination

Amination treatment of HGMs is the core step to achieve in situ polymerization of PPy materials on the surface of HGMs, where amino groups are attached to the surface so that active sites exist on the surface of the inorganic non-metallic material, in order to allow a large number of pyrrole monomers to be oriented and uniformly polymerized on the surface of the HGMs. Silane coupling agent KH550, also known as 3-aminopropyltriethoxysilane, is an organosilicon coupling agent with the chemical formula C_9_H_23_NO_3_Si, which is commonly used to chemically bond with the surfaces of inorganic materials, improve the bonding strength between organic and inorganic materials, and enhance adhesion [37].

**Figure 4:**
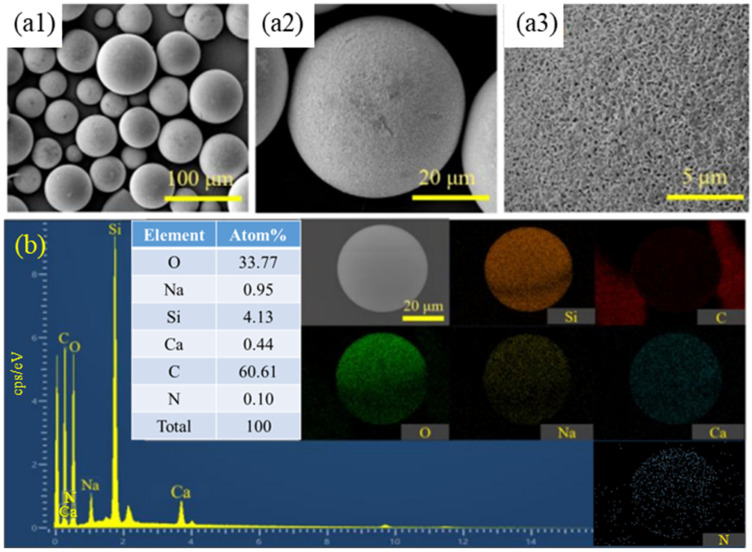
Microscopic characterization of HGMs before and after amination treatment: (**a1**–**a3**) SEM after processing; (**b**) EDS after processing.

As can be seen in Figure 4, the surface of the non-aminated HGMs is slightly rough, which is the result of hydroxylation, and there is also Na_2_SiO_3_ on the surface obtained by the reaction after hydroxylation. In contrast, after the amination treatment, the whole sphere of the HGMs is covered with white material, and there is a dense weave on the surface, which indicates that the silane coupling agent molecules were successfully grafted onto the surface of the glass microspheres. Figure 4b shows the energy spectrum analysis of the swept area of the HGMs after the amination treatment. The EDS energy spectrum analysis of KH550-modified HGMs reveals a considerably higher element C concentration. It can be concluded that the content of organic matter on the surface of the microspheres increased, which suggests that the molecules of the silane coupling agent were grafted onto the surface of the microspheres successfully. From the combined scanning electron microscopy and EDS spectra, the results indicate that the silane coupling agent successfully modified the surface of HGMs.

**Figure 5:**
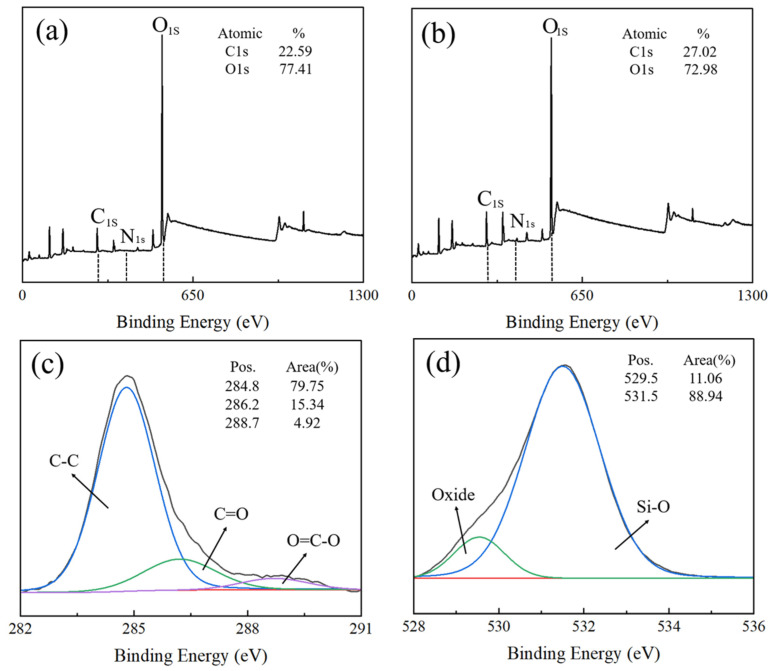
XPS spectra of hollow glass microspheres before and after amination: (**a**) HGMs, (**b**) HGMs after amination, (**c**) C 1s, and (**d**) O 1s.

**Figure 6:**
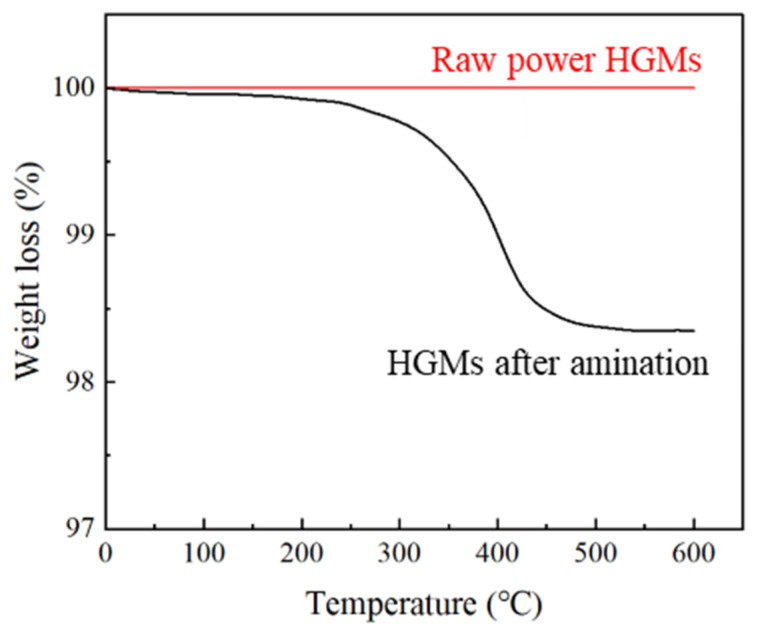
Thermogravimetric curves of hollow glass microspheres before and after amination.

Further investigation of the chemical states of elements C and Si during surface amination of HGMs was conducted using X-ray photoelectron spectroscopic characterization [38]. Figure 5 shows the XPS spectra of the aminated HGMs. Obvious photoelectron spectral peaks of C 1s orbitals and O 1s orbitals can be observed in the full spectrum in Figure 5a,b, and the content of C 1s in the amino-coated HGMs increased from 22.59 atom% to 27.02 atom%, which was the result of the silane coupling agent molecules attaching to the surface of the HGMs. Analyzing the 1s orbitals of C, a clear main peak, representing the C–C bond in the silane coupling agent molecule, appeared at a binding energy of 284.8 eV with a content of 79.75%. A weaker peak appears at the binding energy of 286.2 eV, which is the companion peak of the 284.8 eV peak. It was found that the main forms of the C element are the C–C bond (spectral peak of 284.8 eV) and the C = O bond (spectral peak of 286.2 eV). Based on the area share of the peaks, it can be seen that C–C bonds are in the majority. Analyzing the 1s orbitals of O, it can be seen from Figure 5d that the main forms of O elements present in the HGMs after the amination treatment are Si-O bonds as well as other oxides. Figure 6 shows the heat–weight loss curves of HGMs before and after amination, from which it can be seen that the water content of the raw powder HGMs itself is very low, and when the temperature reaches 600 °C, the quality of the raw powder HGMs is almost unchanged. However, after the treatment with the KH550 silane coupling agent, a layer of organic matter was attached to the surface of the HGMs, which caused the quality of aminated HGMs to decrease at a faster speed after 300 °C, and eventually decrease by about 2.5%. After the treatment of the KH550 silane coupling agent, the surface of HGMs was covered by a layer of organic matter, which caused the quality of aminated HGMs to decrease rapidly after 300 °C, with a final decrease of about 2.7%.

**Figure 7:**
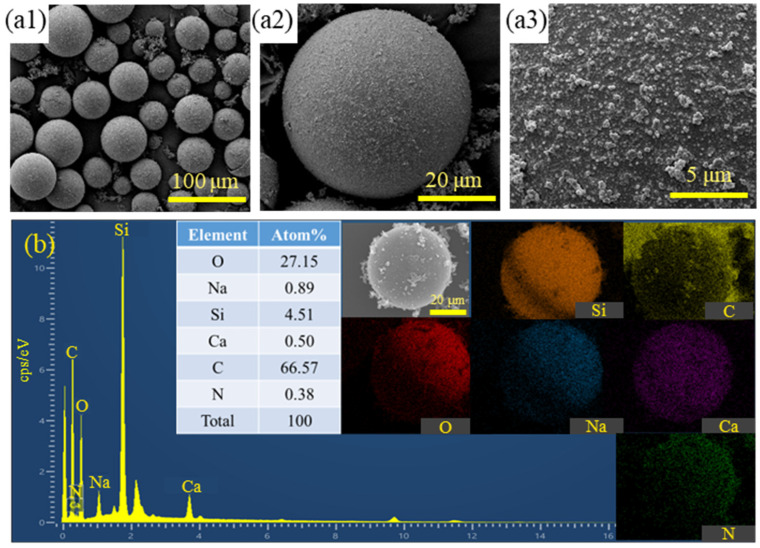
Microscopic characterization of PPy-coated HGMs before and after: (**a1**–**a3**) SEM after coating; (**b**) EDS after coating.

To observe the microscopic morphology and chemical element distribution of PPy-coated hollow microspheres, the microspheres were characterized using scanning electron microscopy (SEM, S-4800, Hitachi, Honshu Island, Japan) and energy dispersive spectroscopy (EDS) before and after coating, as illustrated in Figure 7. The figure reveals that the surface of the HGMs, prior to PPy coating, was dense and relatively flat, with silane coupling agent molecules resulting from the amination treatment. After the PPy coating, the entire surface of the HGMs was covered with a black material, featuring numerous granular coatings ranging from 200 nm to 500 nm in size, with the polymer grains closely packed together. Some particles exhibit a spherical shape, protruding from the surface of the coating layer, indicating a uniform and effective coating distribution. Figure 7b presents the energy spectrum analysis of the area of HGMs after the PPy coating treatment. The results indicate that the elemental carbon content on the surface of the HGMs is approximately 66.57 atom%, confirming that PPy was successfully encapsulated on the surface of the microspheres, with the majority of the material distributed on the surface and a smaller amount located outside the microspheres [39].

**Figure 8:**
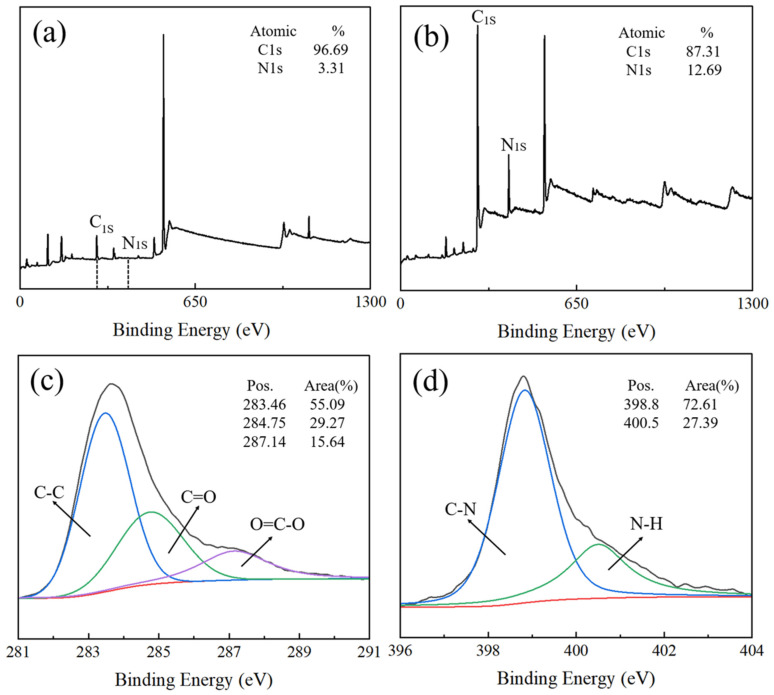
XPS patterns before and after PPy coating of HGMs: (**a**) full spectrum before coating; (**b**) full spectrum after coating; (**c**) C 1s; (**d**) N 1s.

X-ray photoelectron spectroscopy characterization was performed to further investigate the chemical state of elements C and N during the surface coating of HGMs with PPy. Figure 8 shows the XPS spectra of PPy capped on the surface of HGMs. Obvious photoelectron spectral peaks of C1s orbitals and N1s orbitals can be observed in the full spectrum in Figure 8a,b, and the content of N1s in the HGMs after PPy coating treatment was increased from 3.31 atom% to 12.69 atom% with respect to C, which was the result of the PPy attachment to the surface of the HGMs. Analyzing the 1s orbital of C, a clear main peak representing the C-C bond in the silane coupling agent molecule appeared at a binding energy of 283.4 eV with a content of 55.09%. A weaker peak was observed at the binding energy of 284.75 eV, which is the companion peak of the 283.4 eV peak. From the analysis, it was found that the main forms of the C element are the C-C bond (spectral peak of 283.4 eV) and the C=O bond (spectral peak of 284.75 eV). Based on the area share of the peaks, it can be seen that C-C bonds predominate. Analyzing the 1s orbitals of N, it can be seen from Figure 8d that the main forms of N elements in the PPy-treated HGMs are C-N bonds and N-H bonds, which represent the bonding between the silane coupling agent molecules and the PPy molecules. The combination of the SEM, EDS, and XPS results indicates that PPy was successfully encapsulated onto the surface of HGMs.

### 3.2. Influence of Process Parameters on the Surface Effect of HGMs

The morphology, weight gain, and particle size changes of HGM-coated composite powders are critical criteria for evaluating the effectiveness of the coating. The morphology of the powder primarily concerns whether the HGMs are uniformly coated on the surface of the matrix material after in situ polymerization, the homogeneity of the coating layer, and the dispersion of the coated powder. Weight gain serves as an intuitive indicator of the HGM content in the in situ polymerization reaction and reflects the conversion efficiency of the polymerization process. Changes in particle size provide a clear characterization of the thickness of the HGM surface coating layer and the overall coating effectiveness. It is vital to note that changes to the settings in the in situ polymerization process might have a substantial impact on HGM coating performance. The objective is to optimize the coating performance of composite powders, minimize resource consumption, and enhance the feasibility of preparing HGM composite powders.

### 3.3. Influence of Reaction Time on Coating Effect

In the in situ polymerization reaction, the duration of the reaction is a critical influencing factor, as variations in reaction time can significantly impact the final coating quality [40]. This section investigates how changes in reaction duration affect the quality, flotation rate, encapsulation rate, and particle size of HGMs following the in situ polymerization and flotation processes. Additionally, morphological changes on the surface of encapsulated HGMs under different reaction durations were observed using scanning electron microscopy. The reaction conditions outlined in Table 2 were selected for experimental investigation.

**Figure 9:**
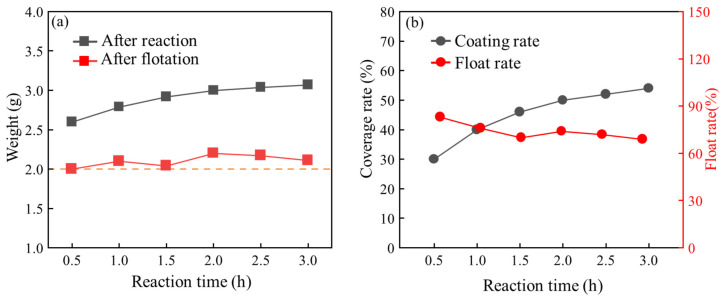
Influence of different reaction times on the coating effect of HGMs: (**a**) quality after reaction and flotation; (**b**) coating rate and flotation rate.

Figure 9 illustrates the impact of varying reaction durations on the coating effectiveness of HGMs. In Figure 9a, the relationship between different reaction durations and the mass of HGMs post-reaction and after flotation is depicted. The initial mass of HGMs was 2.0 g. Following the in situ polymerization reaction, the mass of the composite powders gradually increased with longer reaction times. When the reaction duration exceeded 0.5 h, the mass growth of the microspheres began to plateau. Subsequently, the growth rate of the microspheres decreased every half hour, and after reaching a reaction time of 2.0 h, the mass of HGMs remained nearly constant. This is attributed to the increased likelihood of HGM fragmentation with extended reaction times, resulting in the mass of HGMs after flotation consistently being close to 2.0 g. Figure 9b presents the effects of varying reaction times on the flotation rate and encapsulation rate of HGMs post-reaction. As the reaction time increased, the coating rate of the composite powder also rose. Notably, when the reaction duration reached 0.5 h, the coating rate of HGMs increased rapidly. Beyond this point, the growth rate of the coating rate began to level off, and after 2.0 h, the coating rate of the HGMs remained relatively unchanged. Through testing the reaction time of the microspheres, it was observed that the flotation rate of the microspheres increased with longer reaction times. However, the flotation rate of the reacted microspheres exhibited an overall decreasing trend, dropping from 90% to approximately 70%. This drop can be attributed to the stirring throughout the reaction phase, which contributed to the microspheres’ greater breaking rate.

**Figure 10:**
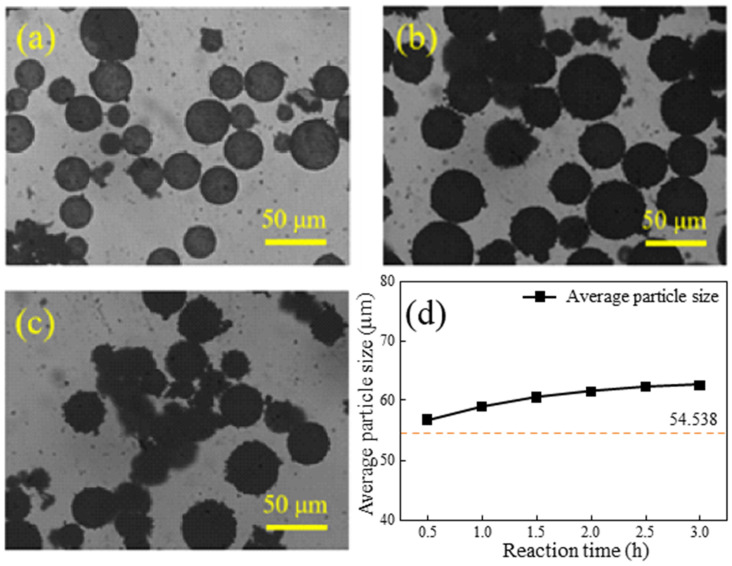
Metallographic microscopy of HGMs at different in situ polymerization reaction times: (**a**) 0.5 h; (**b**) 1.5 h; (**c**) 2.5 h. (**d**) Particle size changes with time.

Figure 10a–c illustrates the metallographic diagrams of HGMs at different reaction lengths, while Figure 10d depicts the relationship between particle size and reaction time. At a reaction time of 0.5 h, as shown in Figure 10a, the HGMs are coated with the substance, although their surfaces remain transparent. After 1.5 h of reaction, the HGMs appear completely black, yet their spherical shape and smooth surface are still discernible. By 1.5 h, larger particles formed on the surface of the HGMs, giving them an overall appearance reminiscent of ‘lychee’. Additionally, as the reaction time increases, the average particle size of the composite powders gradually rises from an initial 54.5 μm to approximately 60.0 μm. This increase in particle size correlates with the change in mass during the reaction. These observations suggest that the in situ polymerization reaction of HGMs primarily occurs in the early stages, with the fragmentation of HGMs progressively increasing as reaction time extends, ultimately leading to a decrease in the flotation rate of the microspheres.

**Figure 11:**
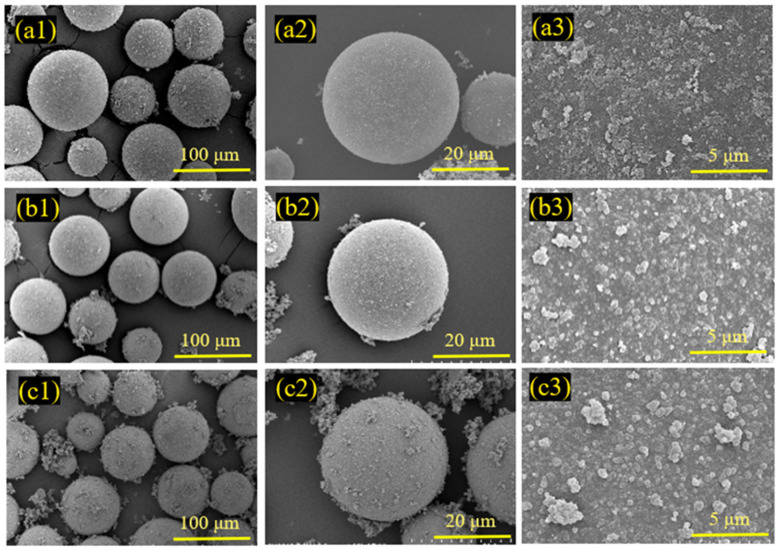
SEM morphology of PPy@HGMs composite powder under different reaction times: (**a1**–**a3**) 0.5 h; (**b1**–**b3**) 1.5 h; (**c1**–**c3**) 2.5 h.

To further investigate the microscopic morphology of the surface of HGMs at varying reaction durations, SEM characterization of the microspheres was conducted, as illustrated in Figure 11. At shorter reaction durations, the amount of HGMs on the surface was minimal, resulting in uncoated areas, and the coated HGM particles were irregular in size and dispersed across the surface. When the reaction duration was extended to 1.5 h, the encapsulation on the surface of HGMs was significantly improved, with a substantial increase in the amount of HGMs present, forming a dense encapsulation network. The edges of the particles appeared more rounded compared to those observed at 0.5 h. Although the encapsulated particles remained small, they completely covered the surface of the HGMs. A careful examination of the boundaries between neighboring particles revealed that the distinction between them was gradually diminishing, suggesting a tendency for the particles to merge and grow, ultimately forming a homogeneous and smooth cladding layer. At a reaction duration of 2.5 h, a fully dense network was established, and numerous PPy agglomerates were observed outside the microspheres. This phenomenon indicates that the HGMs reached surface saturation, and the pyrrole monomer is no longer able to polymerize on the surface of the HGMs.

**Figure 12:**
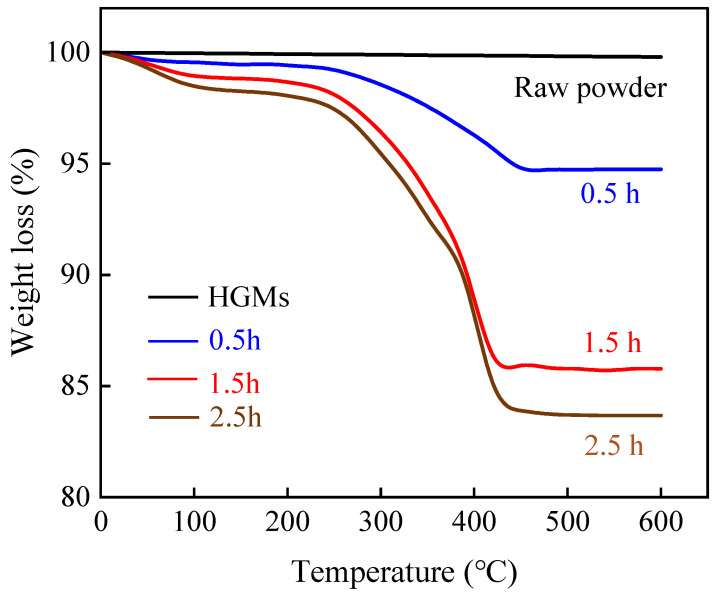
Thermogravimetric curves of HGMs under different reaction times.

Figure 12 illustrates the thermal weight loss curves of HGMs under varying reaction times. Pure HGMs did not decompose at high temperatures, remaining stable up to 600 °C. In contrast, the decomposition of PPy-coated HGMs commenced around 50 °C, with the rate of decomposition gradually accelerating around 250 °C. Complete decomposition occurred around 430 °C, resulting in a mass loss of approximately 17.1% for the modified HGMs after a reaction time of 2.5 h. When comparing different reaction times, it is evident that the mass loss of HGMs after 1.5 h was significantly lower than that observed after 0.5 h. This indicates that the polymerization reaction of the pyrrole monomer predominantly occurs during this stage.

### 3.4. Influence of Microsphere/Pyrrole Mass Ratio on Coating Effect

**Figure 13:**
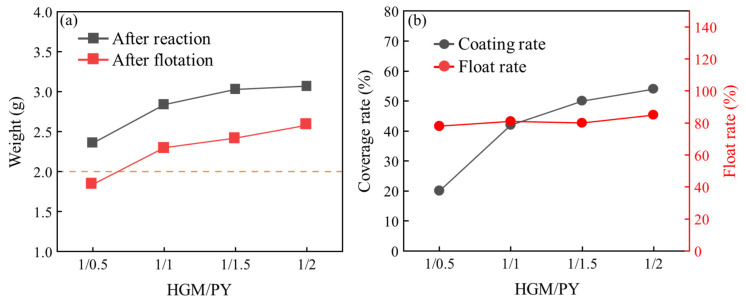
Influence of different HGMs/PPy quality ratios on coating effect: (**a**) quality after reaction and flotation; (**b**) coating rate and flotation rate.

The varying mass ratios of microspheres to pyrrole significantly influence the final coating quality. In this study, we investigated the effects of different microsphere/pyrrole mass ratios during the reaction process, focusing on how reaction time impacts the quality, floating rate, coating rate, particle size, and distribution of HGMs during in situ polymerization. We also observed the morphological changes on the surface of HGMs coated at varying reaction times using scanning electron microscopy. The process parameters outlined in Table 3 were selected for the in situ polymerization experiments. Figure 13 illustrates the impact of different microsphere/pyrrole mass ratios on the coating effectiveness of HGMs. As shown in Figure 13a, the mass of the composite powders increased progressively with higher microsphere/pyrrole mass ratios. Notably, the mass of HGMs surged from an initial 2.0 g to approximately 2.4 g when the microsphere/pyrrole mass ratio reached 1.0:0.5. Beyond this point, the mass growth of the microspheres began to plateau, and the growth rate gradually diminished. After the microsphere/pyrrole mass ratio reached 1.0:1.5, the mass of HGMs exhibited minimal change. The mass of HGMs post-flotation demonstrated a consistent upward trend, with the rate of increase mirroring that of the mass after the reaction, indicating that the breakage rate of HGMs remained relatively constant across the same reaction time. Figure 13b depicts the influence of varying microsphere/pyrrole mass ratios on the floating rate and encapsulation rate of HGMs following the reaction. As the microsphere/pyrrole mass ratio increased, the coating rate of the composite powders rose steadily. The HGM coating rate experienced a rapid increase at a microsphere/pyrrole mass ratio of 1:1, but the growth of the coating rate began to level off when the ratio exceeded 1:1. The coating rate of HGMs showed little variation once the microsphere/pyrrole mass ratio reached 1:2. Furthermore, testing of the flotation rate of the microspheres after the reaction revealed that, despite the increase in the mass ratio of microspheres to pyrrole, the floating rate of HGMs remained relatively stable at around 88.0%. This stability can be attributed to the unchanged reaction time and the minimal variation in the breakage rate of the microspheres.

**Figure 14:**
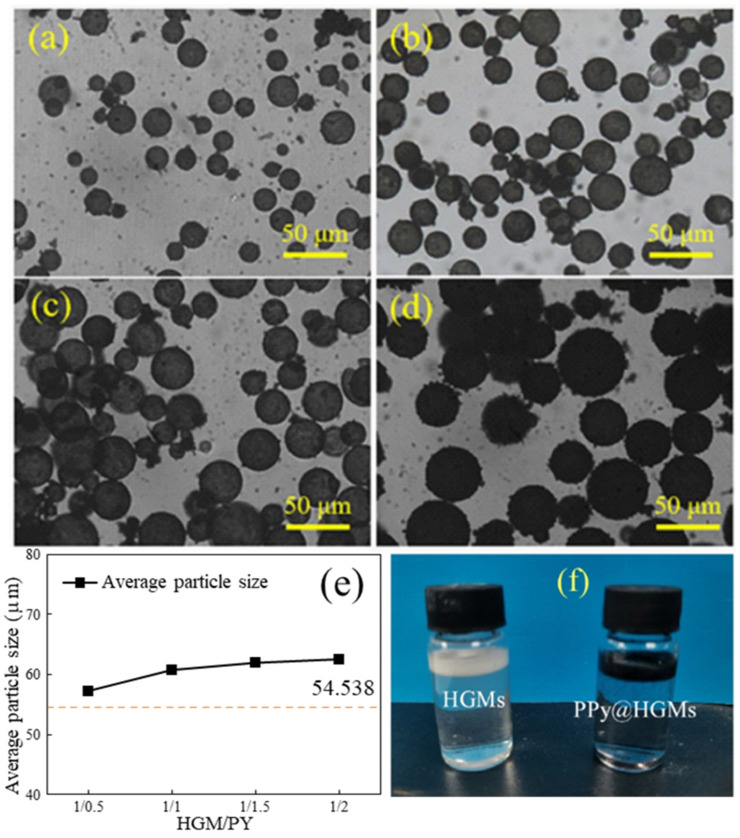
Metallographic microscope images of HGMs with different HGMs/PPy mass ratios: (**a**) 1/0.5; (**b**) 1/1; (**c**) 1/1.5; (**d**) 1/2. (**e**) Influence of the mass ratio of different microspheres/pyrrole on the particle size of HGMs. (**f**) Photograph of HGMs and PPy@HGMs.

Figure 14 presents the metallographic microscope images of HGMs at various microsphere/pyrrole mass ratios. It is evident that when the microsphere/pyrrole mass ratio is 1:0.5, as illustrated in Figure 14a, some substances appear on the surface of the HGMs, while the overall state remains transparent. At a microsphere/pyrrole mass ratio of 1:1, the surface of the HGMs exhibits a deeper black coloration, although the transparent state of the HGMs is still discernible. When the microsphere/pyrrole mass ratio increases to 1:2, the black surface of the HGMs becomes completely enveloped, and larger HGM particles emerge on the surface, rendering the microspheres entirely invisible in the transparent state. From Figure 14e, it is observed that as the mass ratio of microspheres to pyrrole increases, the average particle size of the composite powders gradually rises from an initial 54.5 μm to approximately 60.0 μm, with the rate of increase gradually slowing down. This phenomenon is primarily attributed to the saturating effect on the surface of the HGMs, which prevents the pyrrole monomer from polymerizing on the microspheres’ surface in the later stages. Figure 14f illustrates the macroscopic morphology before and after the HGM coating process. The raw HGM powder is a white powder with good fluidity and a density of around 0.4 g/cm^3^, allowing it to float on the water’s surface. Following the HGM coating, the material transitions from a white powder with good fluidity to a black powder, which continues to float on the water’s surface. This indicates that the HGMs modified by the coating process still retain their lightweight properties.

**Figure 15:**
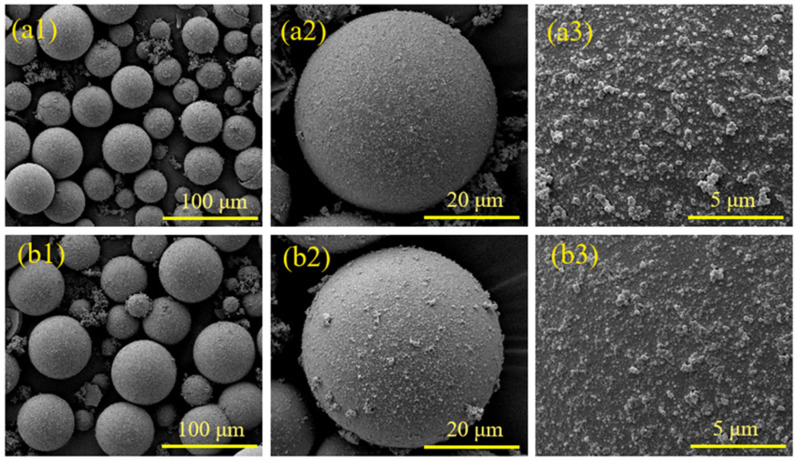
SEM diagram of HGMs under different HGMs/PPy mass ratios: (**a1**–**a3**) 1/1; (**b1**–**b3**) 1/2.

The HGMs obtained at different microsphere/pyrrole mass ratios were analyzed using SEM to observe the encapsulation of HGMs on the surfaces, as illustrated in Figure 15(a1–b3). These figures depict the microscopic morphology of PPy@HGMs at microsphere/pyrrole mass ratios of 1:1 and 1:2, respectively. At a microsphere/pyrrole mass ratio of 1:1, the HGMs on the surface, as shown in Figure 15(a1–a3), completely encapsulated the HGMs, resulting in a more uniform dispersion of the encapsulated HGMs particles. Notably, larger HGM particles are absent. In contrast, at a mass ratio of 1:2, the surface coating of HGMs in Figure 15(b1–b3) appears significantly denser, with a substantial increase in the number of HGMs forming a robust coating network. This configuration includes numerous HGMs both on the surface of the microspheres and outside of them, leading to the formation of a completely dense network devoid of prismatic corners. Additionally, there are many agglomerates of HGMs outside the microspheres, indicating that the surface of the polymerized HGMs reached saturation.

**Figure 16:**
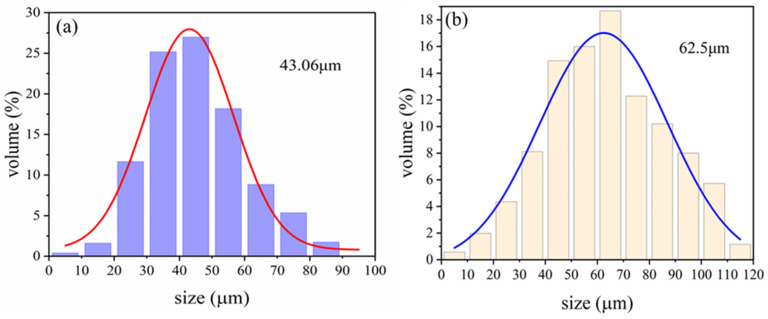
Particle size distribution of (**a**) HGMs; (**b**) PPy-coated HGMs (PPy content: 50%).

Figure 16 illustrates the particle size distribution of HGMs and HGMs coated with 50% PPy. The particle size distribution of the HGM powder is broad, exhibiting a normal distribution between 1 and 90 μm, with the majority of the microspheres concentrated in the 20–60 μm range and an average size of 43.06 μm. In contrast, the size of the PPy@HGMs particles is approximately normally distributed within the range of 5–120 μm, with the highest proportion of particles falling within the 60–70 μm range, accounting for 18.8%. When the PPy coating amount is 50%, the average particle size of the HGMs is calculated to be 62.5 μm, representing a 45.15% increase compared to the original powder.

## 4. PPy@HGMs Surface Coating Mechanisms

Scanning electron microscopy was used to examine HGMs following each polypyrrole in situ polymerization coating procedure. HGM powder as a whole after multiple changes in the process treatment showed no particularly obvious changes; hydroxylation and amination result in some white material being attached to the surface of the HGMs, but in situ polymerization of pyrrole produces some particulate matter on the surface of the HGMs. Selected single microspheres for scanning electron microscopy study may clearly detect the changes in the surface of the HGMs, from the smooth surface at the beginning to later becoming increasingly rougher, as well as an increase in organic matter on the surface. Following the nitrogen-based modification treatment, the surface of the HGMs fully adhered to a layer of white material, and the coating became denser. The coating of the HGM surface after the poly-tetragram changed into a black material, and the surface of the material was comparatively rougher. Flotation occurs when the surface of the HGMs only contains a small amount of white particles and small particle sizes. This is caused by the reaction with NaOH solution to produce Na_2_SiO_3_ material. Following flotation, a small number of particles with a size of approximately 100 nm were present on the surface; following hydroxylation treatment, the HGMs’ surface had flakes of Na_2_SiO_3_; following amination modification treatment, the HGMs’ surface contained a dense weaving substance, which is the silane coupling agent that was successfully attached to the HGMs’ surface; and following polybrene coating, the surface of the HGMs had a large number of black ball particles that aggregated together. This results from the pyrrole monomer successfully polymerizing into polypyrrole on the surface of HGMs.

**Figure 17:**
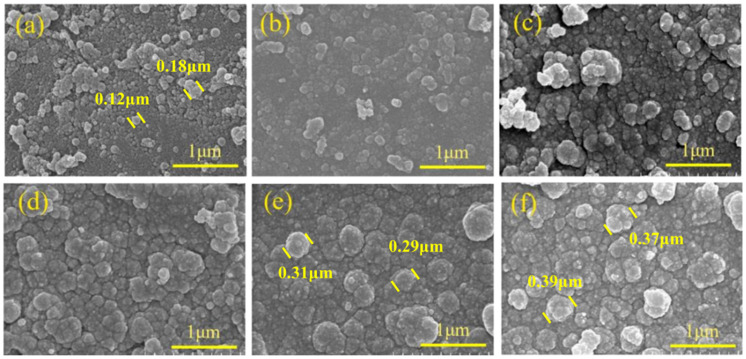
Surface morphology of PPy@HGMs under different reaction times: (**a**) 0.5 h; (**b**) 1.0 h; (**c**) 1.5 h; (**d**) 2.0 h; (**e**) 2.5 h; (**f**) 3.0 h.

The surface morphology of HGMs during various stages of the in situ polymerization reaction was examined using scanning electron microscopy. This analysis aimed to elucidate the polymerization process of PPy on the surface of the HGMs and to investigate the growth mechanism of PPy. As illustrated in Figure 17, during the in situ polymerization reaction, the amino functional groups on the surface of the HGMs create activation centers that attract pyrrole monomers from the reaction solution. These monomers deposit and grow at the activation centers. As the reaction progresses, the deposition rate of pyrrole monomers in the plating solution accelerates, leading to further polymerization among the monomers. Consequently, the grains continue to grow larger and expand outward, causing adjacent particles to merge and form larger grains [41,42]. After that, the pyrrole monomer continues to polymerize on the surface of the HGMs, promoting the growth of the PPy chains until the surface is completely saturated.

**Figure 18:**
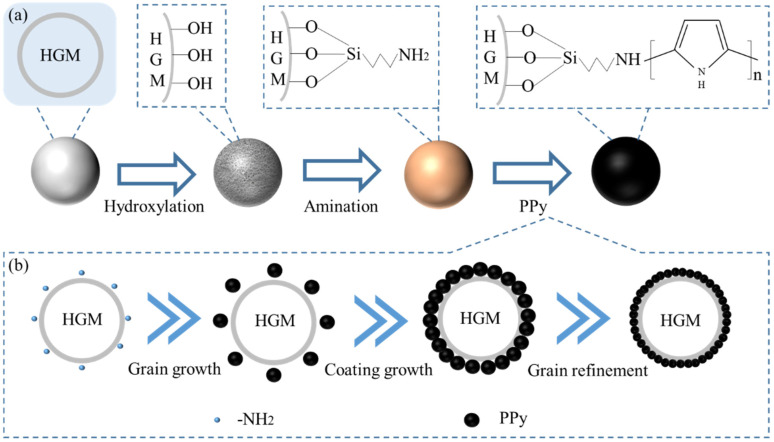
Mechanism diagram of PPy-coated HGMs: (**a**) entire process; (**b**) polymerization and encapsulation of pyrrole on the surface of HGMs.

Figure 18 illustrates the mechanism of PPy-coated HGMs. Initially, the HGMs undergo flotation and hydroxylation treatments to eliminate defective microsphere powder, resulting in a surface abundant in hydroxyl functional groups. Subsequently, an amination treatment is applied, which attaches a membrane layer containing amino groups to the surface of the HGMs. This layer provides active sites for the subsequent polymerization of pyrrole monomers. The amino functional groups on the surface of the HGMs exhibit nucleophilic and electrophilic interactions with the sub-amino groups in the pyrrole ring through electron-donating effects, thereby attracting the pyrrole monomer to deposit and grow at these active sites. The pyrrole monomer is oxidized to form free cationic radicals, which couple to form a dimer, releasing protonated hydrogen to yield a neutral dimer. This neutral dimer is then oxidized to form dimeric radicals, which react with other monomers, dimers, and oligomer radicals, driving the growth of the polymer chain and resulting in the formation of the conjugated chain of PPy [43]. In the interaction between PPy and the amino groups on the surface of HGMs, the Lewis acid characteristics of PPy react strongly with the Lewis-type electron-donating amino groups in KH550. This reaction results in robust bonding of the products and the formation of an effective interface [44].

## 5. Conclusions

(1) The development of a new process that integrates flotation, hydroxylation, amination pretreatment, and in situ surface polymerization is a significant advancement. This integrated approach addresses the challenges associated with the coating of HGMs, which is a complex task due to their low density, high specific surface area, thin cavity shells, and poor surface adhesion.
(2) Achieving a uniform coating of PPy on HGMs is a notable accomplishment. This uniformity is crucial for the performance of HGMs in various applications, as it ensures consistent properties across the material.
(3) Flotation treatment can effectively remove defective particles in HGMs and reduce their density. The density of HGMs decreased from 0.3841 g/cm^3^ to 0.3111 g/cm^3^ after flotation.
(4) Different reaction durations and microsphere/PPy mass ratios in the in situ polymerization reaction influence the extent of PPy encapsulation and the size of the encapsulated particles. The coated HGMs retained their lightweight properties. The in situ polymerization resulting in a uniform encapsulation of spherical PPy, with an average particle size increase of 14.60%, is a significant result. This indicates that the coating process not only improves the functionality of HGMs but also enhances their physical properties.
(5) The mechanism of PPy coating on HGMs: The amino groups on the surface of HGMs attract pyrrole, leading to the enrichment of pyrrole and promoting the growth of the PPy chain. This process contributes to the homogeneous dispersion of PPy on the surface of HGMs. The Lewis acid properties of PPy react strongly with the Lewis-type electron-donating amino groups in the silane coupling agent, enhancing the bonding strength of the product and facilitating the formation of a robust interface.

## Figures and Tables

**Table 1:** Concentration of the used solution.

Number	Reagent Name	Chemical Formula	Concentration
1	KH550	C_9_H_23_NO_3_Si	1%
2	Ethyl alcohol	C_2_H_5_OH	24%
3	Deionized water	H_2_O	75%
4	5-Sulfosalicylic acid	C_7_H_6_O_5_S	1.0 mol/L
5	Ethyl alcohol	C_2_H_5_OH	7.0%
6	Deionized water	H_2_O	93.0%
7	Pyrrole	C_4_H_5_N	1.0–4.0 g
8	Ferric chloride	FeCl_3_	1.0 mol/L

**Table 2:** In situ polymerization reaction process parameters under different reaction times.

Number	Technological Parameter	Numerical Value
1	HGMs/pyrrole	1:2
2	Water:ethyl alcohol	10:1
3	5-Sulfosalicylic acid concentration	1 mol/L
4	Fecl_3_ concentration	1 mol/L
5	Temperature	0 °C
6	Reaction time	0.5–3.0 h

**Table 3:** Process parameters of polymerization under different mass ratios of HGMs/PY.

Number	Technological Parameter	Numerical Value
1	HGMs/pyrrole	0.5–2.0
2	Water:ethyl alcohol	10:1
3	5-Sulfosalicylic acid concentration	1 mol/L
4	Fecl_3_ concentration	1 mol/L
5	Temperature	0 °C
6	Reaction time	3.0 h

## Data Availability

The original contributions presented in the study are included in the article/Supplementary Materials, further inquiries can be directed to the corresponding authors.

## Supplementary Materials

The following supporting information can be downloaded at: https://www.mdpi.com/article/10.3390/ma17225595/s1, Figure S1 SEM microscopic characterization of HGMs raw powder. Figure S2 SEM images of HGMs before and after flotation: (a1,a2) before flotation; (b1,b2) after flotation.

## References

[1] Wang H., Zheng K., Zhang X., Wang Y., Xiao C., Chen L., Tian X. (2018). Hollow microsphere-infused porous poly (vinylidene fluoride)/multiwall carbon nanotube composites with excellent electromagnetic shielding and low thermal transport. J. Mater. Sci..

[2] Chen W., Qin Y., He X., Su Y., Wang J. (2022). Light-weight carbon fiber/silver-coated hollow glass spheres/epoxy composites as highly effective electromagnetic interference shielding material. J. Reinf. Plast. Compos..

[3] Wang Y., Chen S., Liu F., Dang Z. (2024). Experimental and Computational Study of the Thermal Insulation Properties of Hollow Glass Microspheres. Energy Technol..

[4] Song L., Zong L.-S., Wang J.-Y., Jian X.-G. (2021). Preparation and Performance of HGM/PPENK-based High Temperature-resistant Thermal Insulating Coatings. Chin. J. Polym. Sci..

[5] Wang L., Cui H., Han X., Wang X. (2021). Floating microparticles of ZnIn_2_S_4_ @hollow glass microsphere for enhanced photocatalytic activity. Int. J. Hydrogen Energy.

[6] Huang Z., Chi B., Guan J., Liu Y. (2014). Facile method to synthesize silver nanoparticles on the surface of hollow glass microspheres and their microwave shielding properties. RSC Adv..

[7] Wang P., He B., An Z., Xiao W., Song X., Yan K., Zhang J. (2024). Hollow glass microspheres embedded in porous network of chitosan aerogel used for thermal insulation and flame retardant materials. Int. J. Biol. Macromol..

[8] Qiu R., Wang B., Shang J., Hu G., Yu L., Gao X. (2024). Modifying hollow glass microspheres to obtain self-floating separation adsorbents for adsorbing pollutants in wastewater: A review. J. Mol. Liq..

[9] Yang J., Jeon D., Kang H., Shang X., Moon J. (2023). Hydrophobic treatment on hollow glass microspheres for enhancing the flowability of lightweight high-performance cementitious composites. Constr. Build. Mater..

[10] Zhao K., Liu H., Wang T., Zeng H. (2016). Cu-plated hollow glass microspheres for hydrogen production and degradation. J. Mater. Sci. Mater. Electron..

[11] Niazi P., Karevan M., Javanbakht M. (2023). Mechanical and thermal insulation performance of hollow glass microsphere (HGMs)/fumed silica/polyester microcomposite coating. Prog. Org. Coat..

[12] Yu Z., Du X., Zhu P., Zhao T., Sun R., Chen J., Wang N., Li W. (2022). Surface modified hollow glass microspheres-epoxy composites with enhanced thermal insulation and reduced dielectric constant. Mater. Today Commun..

[13] Mahmoud M., Kraxner J., Elsayed H., Bernardo E., Galusek D. (2023). Fabrication and environmental applications of glass microspheres: A review. Ceram. Int..

[14] Wang X., Zeng L., Liu W., Qiao Y., Zhang L., Bai C., Su S., Shen J., Zheng T. (2024). Constructing ‘bayberry shape’ structure on HGMs surface using CNTs to enhance the mechanical properties of HGMs/epoxy composites. Compos. Commun..

[15] Afolabi O.A., Kanny K., Mohan T.P. (2021). Processing of hollow glass microspheres (HGMs) filled epoxy syntactic foam composites with improved structural characteristics. Sci. Eng. Compos. Mater..

[16] Zhao X., Wang H., Peng H., Wang L., Lu X., Huang Y., Chen J., Shao T. (2017). Buoyant ALG/HA/HGMs composite adsorbents for highly efficient removal of copper from aqueous solution and contaminated kaolin soil. Chem. Eng. J..

[17] Zeng L.W., Bian J.J. (2023). Ultralow-k hollow glass microsphere filled perfluoroalkoxy composites. Int. J. Appl. Ceram. Technol..

[18] An Y., Zheng P., Ma X. (2019). Preparation and visible-light photocatalytic properties of the floating hollow glass microspheres-TiO_2_/Ag_3_PO_4_ composites. RSC Adv..

[19] Zhang H. (2013). Silver plating on hollow glass microsphere and coating finishing of PET/cotton fabric. J. Ind. Text..

[20] Kanwal R., Maqsood M.F., Raza M.A., Inam A., Waris M., Rehman Z.U., Mehdi S.M.Z., Abbas N., Lee N. (2024). Polypyrrole coated carbon fiber/magnetite/graphene oxide reinforced hybrid epoxy composites for high strength and electromagnetic interference shielding. Mater. Today Commun..

[21] Chen X., Qi S. (2016). Synthesis and microwave absorption enhancement of polyaniline/SrFe_12_O_19_/hollow glass microsphere composite with core-shell structure. J. Mater. Sci. Mater. Electron..

[22] Xu R., Wang W., Yu D. (2019). Preparation of silver-plated Hollow Glass Microspheres and its application in infrared stealth coating fabrics. Prog. Org. Coatings.

[23] Chen Q., Han B. (2018). Microporous Polycarbazole Materials: From Preparation and Properties to Applications. Macromol. Rapid Commun..

[24] Li X., Xue Y., Zhang D., Chen Y. (2023). Robust silicone rubber with high transparency by loading with porous hollow glass microsphere. J. Polym. Res..

[25] Wang Y.-Y., Zhang F., Li N., Shi J.-F., Jia L.-C., Yan D.-X., Li Z.-M. (2023). Carbon-based aerogels and foams for electromagnetic interference shielding: A review. Carbon.

[26] Huo S., Wang J., Wu X. (2017). Morphology, thermal and mechanical performances of SR composites containing sepiolite and HGMs as binary fillers. J. Polym. Eng..

[27] Yang J., Liao X., Wang G., Chen J., Guo F., Tang W., Wang W., Yan Z., Li G. (2020). Gradient structure design of lightweight and flexible silicone rubber nanocomposite foam for efficient electromagnetic interference shielding. Chem. Eng. J..

[28] Bu F., Song P., Liu Y., Wang J., Wu X., Liu L., Xu C., Zhang J. (2022). The Effective Surface Metallization of Hollow Glass Microspheres for Flexible Electromagnetic Shielding Film. J. Wuhan Univ. Technol. Sci. Ed..

[29] Krakowiak K.J., Nannapaneni R.G., Moshiri A., Phatak T., Stefaniuk D., Sadowski L., Qomi M.J.A. (2020). Engineering of high specific strength and low thermal conductivity cementitious composites with hollow glass microspheres for high-temperature high-pressure applications. Cem. Concr. Compos..

[30] Imran M., Pal S., Kahaly M.U., Rahaman A. (2021). Radially grown carbon nanomaterials on hollow glass microspheres and their application in composite foams with excellent electromagnetic interference shielding. Polym. Compos..

[31] Bu F., Zhang J., Yu S., Li Q., Li G., Wang J., Wu X., Goto T. (2020). Effective surface pretreatment of hollow glass microspheres via a combined KF roughening and alkali washing strategy for the following metallization. Adv. Powder Technol..

[32] Kang S., Hong S.I., Choe C.R., Park M., Rim S., Kim J. (2001). Preparation and characterization of epoxy composites filled with functionalized nanosilica particles obtained via sol–gel process. Polymer.

[33] Dai P., Tao M., Li X., Xue Z., Zeng J., Chen L. (2024). Effect of lime (CaOH^+^) on chalcopyrite-arsenopyrite separation in high gradient magnetic separation: Experiment and molecular simulation. Sep. Purif. Technol..

[34] Yan K., Xie X., Li B., Yuan J., Zhang J. (2011). Influence of the real density and structure imperfection of hollow glass microspheres on the compression strength. Mater. Sci. Eng. A.

[35] Shao X., Han S., Kang Y., Yang X., Yang L., Zhang Q., Wang X. (2024). The plasma-coupling agent synergistically modified the acoustic matching layer for the preparation of a gas ultrasonic flowmeter. Mater. Sci. Semicond. Process..

[36] Sai B.K., Tambe P. (2022). Surface modified hollow glass microsphere reinforced 70/30 (wt/wt) PC/ABS blends: Influence on rheological, mechanical, and thermo-mechanical properties. Compos. Interfaces.

[37] Wang P., Zhong S., Yan K., Liao B., Guo Y., Zhang J. (2024). Effect of hollow glass microspheres surface modification on the compressive strength of syntactic foams. J. Mater. Res. Technol..

[38] Ma Y., Du Y., Zhao J., Yuan X., Hou X. (2020). Preparation and Characterization of Furan–Matrix Composites Blended with Modified Hollow Glass Microsphere. Polymers.

[39] Weng Y., Jin Z., Xie S., Zhang M. (2024). Integration of hierarchical magnesium silicate with polypyrrole-coated hollow Fe_3_O_4_ hybrids as a synergistic adsorbent toward efficient water treatment. Colloids Surfaces A Physicochem. Eng. Asp..

[40] Chen Y., Shen L., Wang C., Feng S., Zhang N., Xiang S., Feng T., Yang M., Zhang K., Yang B. (2020). Utilizing in-situ polymerization of pyrrole to fabricate composited hollow nanospindles for boosting oxygen evolution reaction. Appl. Catal. B Environ..

[41] Ren J., Wang C., Zhang H., Liu X., Yang T., Zheng W., Li T., Ma Y. (2023). Magnetic Core@Shell Fe_3_O_4_@Polypyrrole@Sodium Dodecyl Sulfate Composite for Enhanced Selective Removal of Dyestuffs and Heavy Metal Ions from Complex Wastewater. Langmuir.

[42] Faisal M., Ahmed J., Algethami J.S., Jalalah M., Alsareii S.A., Alsaiari M., Harraz F.A. (2023). Visible-light responsive Au nanoparticle-decorated polypyrrole-carbon black/SnO_2_ ternary nanocomposite for ultrafast removal of insecticide imidacloprid and methylene blue. J. Ind. Eng. Chem..

[43] Xie X., Zhou X. (2011). Well encapsulated hollow borosilicate glass sphere@polypyrrole composite with low density, designable thickness and conductivity. Colloids Surfaces A Physicochem. Eng. Asp..

[44] Perruchot C., Chehimi M.M., Delamar M., Cabet-Deliry E., Miksa B., Slomkowski S., Khan M.A., Armes S.P. (2000). Chemical deposition and characterization of thin polypyrrole films on glass plates: Role of organosilane treatment. Colloid Polym. Sci..

