# Supplementary material for: Formation Mechanism of Polypyrrole-Coated Hollow Glass Microspheres (PPy@HGMs) Composite Powder
Source: Materials (Basel). 2024 Nov 15;17(22):5595. doi: 10.3390/ma17225595 (PMC11595547; doi:10.3390/ma17225595)
Supplement: Supplementary file 1 [file materials-17-05595-s001.zip › materials-3248251-supplementary.pdf]

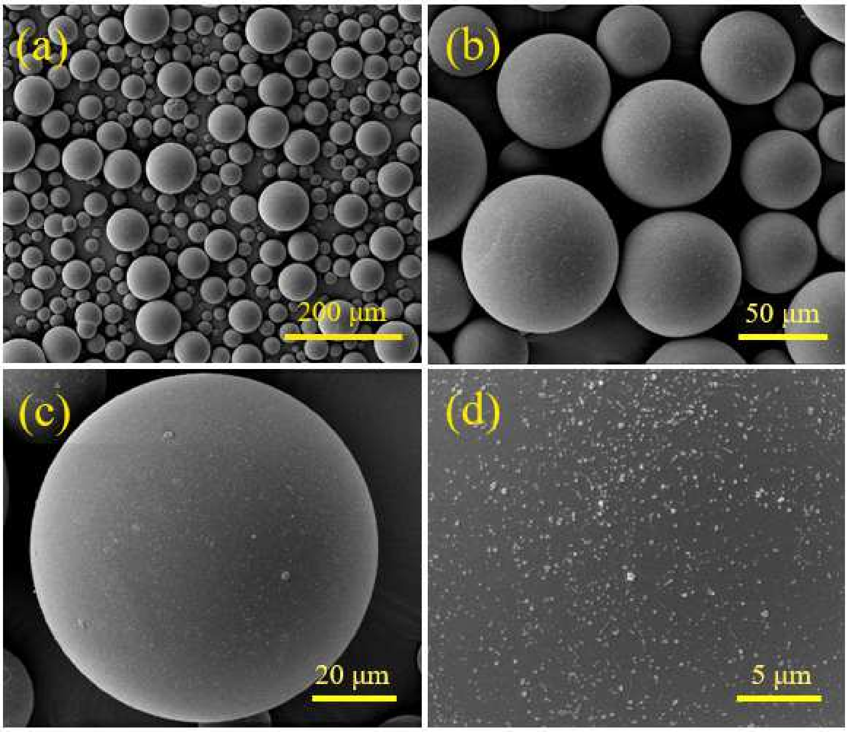

Figure S1 SEM microscopic characterization of HGMs raw powder

SEM and EDS were used to characterize the micro-morphology and analyze the elemental content of the hollow glass microsphere. Figure S1 shows that most of the hollow glass microsphere are regular spherical particles of different sizes, which is consistent with the test results of the size and distribution of the microspheres. In addition to the regular spherical microspheres in Figure S1 (a), there are still some microspheres with incomplete foaming, broken, holes and other defects, which is the reason for the low floating rate of hollow glass microspheres. By increasing the magnification of scanning electron microscope, Figure S1 (c) and (d), it can be found that the surface of the original powder microspheres is relatively smooth, and there are only some fine white particulate matter, which is not conducive to the attachment of active groups and the polymerization of pyrrole monomer, so it is necessary to coarsen the hollow glass microspheres.

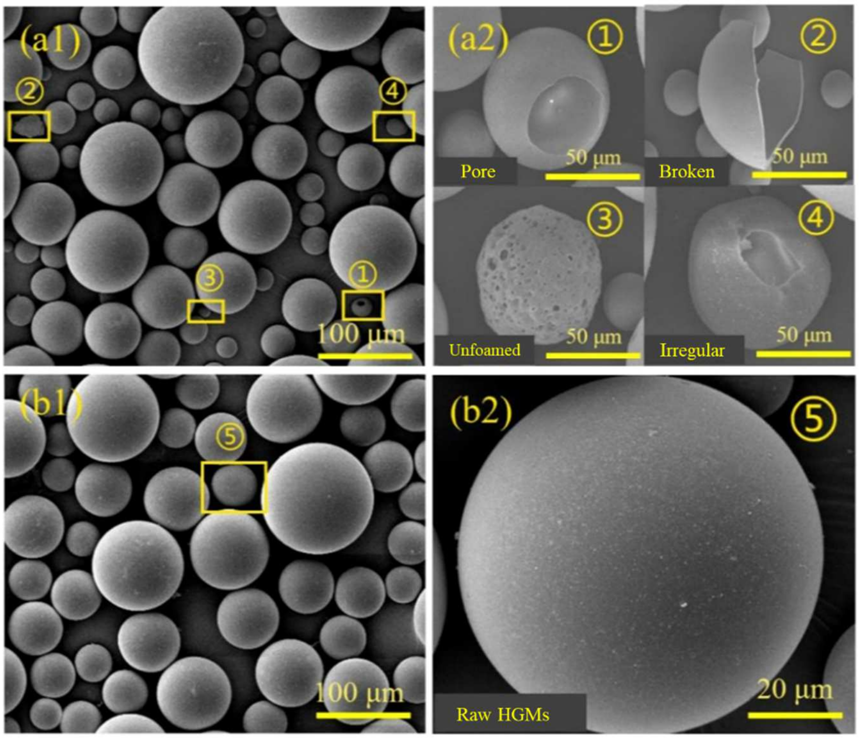

Figure S2 SEM images of HGMs before and after flotation: (a1-a2) before flotation; (b1-b2) after flotation

SEM was used to characterize the morphology of hollow glass microsphere before and after flotation, as shown in Figure S2. From Figure S2 (a1) and (b1), it can be found that after the hollow glass microsphere were flown, the small size and defective microspheres basically disappeared, because the un-foamed small size microspheres and defective microspheres had a high density and sank underwater. The flotation process is to remove all the underwater microspheres, leaving the regular shape and full of hollow glass microsphere spherical particles. Figure S2 (a2) and Figure S2 (b2) mainly characterize the microscopic morphology of defective and normal microspheres, common defective hollow glass microsphere are mainly characterized by incomplete foaming, holes, crushing, irregular shape, etc. The causes of the defective microspheres may be the raw materials, production process, temperature, equipment and other issues. The different defective types of microspheres characterized in Figure S2 (a2) are also reflected in Figure S2 (a1).
